# No difference in mobile and fixed bearing partial knee arthroplasty in octogenarians: a clinical trial

**DOI:** 10.1007/s00590-023-03537-7

**Published:** 2023-04-05

**Authors:** Riccardo D’Ambrosi, Federico Valli, Alessandro Nuara, Ilaria Mariani, Fabrizio Di Feo, Nicola Ursino, Matteo Formica, Laura Mangiavini, Michael Hantes, Filippo Migliorini

**Affiliations:** 1 IRCCS Istituto Ortopedico Galeazzi, Milan, Italy; 2 Department of Biomedical Sciences for Health, University of Milan, Milan, Italy; 3 Institute for Maternal and Child Health IRCCS Burlo Garofolo, Trieste, Italy; 4 Orthopaedic Clinic, IRCCS Hospital Policlinico San Martino, Genoa, Italy; 5 DISC - Department of Surgical Sciences and Integrated Diagnostics, University of Genoa, Genoa, Italy; 6 Department of Orthopaedic Surgery, Faculty of Medicine, University of Thessalia, University Hospital of Larissa, Larissa, Greece; 7 Department of Orthopaedic, Trauma, and Reconstructive Surgery, RWTH University Hospital, 52074 Aachen, Germany

**Keywords:** Knee, Arthroplasty, Mobile-bearing, Fixed-bearing, Octagenarians

## Abstract

**Background:**

A clinical trial comparing MB (mobile-bearing) versus FB (fixed-bearing) in medial partial knee arthroplasty (PKA) in octagenarians has been conducted. The focus of the present study was on PROMs, range of motion (ROM), implant positioning and implants survivorship. The hypothesis of the present study was that MB implants performed better than FB in PKA in octogenarians.

**Methods:**

The first group received FB PKA-PPK®; the second received MB PKA-Oxford. Patients were not randomly allocated. The following PROMs were administered at *T*_0_ (pre-operatively), *T*_1_ (1 year after surgery), and *T*_2_ (3 years after surgery): visual analogue scale (VAS), Knee Society Score (KSS) and Oxford Knee Score (OKS). Data regarding the implant survivorship and ROM were also collected. Furthermore, the following radiographic parameters were measured: Femoral component varus/valgus; Tibial component varus/valgus; Anteroposterior slope.

**Results:**

At *T*_0_, 28 patients were included in the FB and 33 in the MB group. The surgical time was shorter in the FB group (*p* < 0.001). No difference between FB and MB in ROM, VAS, KSS, and OKS at each follow-up (*p* > 0.05). No difference was found in implant positioning (*p* > 0.05). At last follow-up, FB group reported three failures caused by aseptic loosening. Four failures were observed in the MB cohort: two for bearing dislocation and two for aseptic loosening. The Kaplan–Meier Curve found no differences in implant survivorship.

**Conclusions:**

According to the main findings of the present clinical trial, MB implants performed similar to FB in PKA in octogenarians. The FB group demonstrated shorted surgical time. No difference was found in patient reported outcome measures, ROM, implant positioning, and survivorship.

**Level of evidence:**

Level II, prospective study.

## Introduction

From an historical perspective, the concept of an ageing population is a relatively new problem. The World Health Organisation (WHO) estimated that by the year 2050, thirty-eight percent of the world population will be older than 65, and there will be a larger number of elderlies than adolescents [1]. Osteoarthritis of the knee is common in the elderly, impacting negatively the quality of life and participation in recreational activities of affected patients. Osteoarthritis of the knee is a major burden worldwide, with a prevalence of around 10% in men and 20% in women aged 45 years and above in the Caucasian population [2]. In patients with advanced to severe knee osteoarthritis, total knee arthroplasty is advocated [3]. However, in approximately 30% of affected patients, only the medial compartment of the knee is affected, and a partial knee arthroplasty (PKA) may be recommended [4]. PKA preserves the cruciate ligaments and all structures of the healthy compartments. Previous meta-analyses reported that PKA promotes higher patient reported outcome measures (PROMs) compared to total knee arthroplasty [5], along with faster surgical duration, lower estimated blood lost and quicker hospitalization length. Kozinn and Scott in 1989 firstly defined the indications to perform a PKA [6]: monocompartimental osteoarthritis with efficient anterior cruciate ligament, varus deformity less than 5°, minimum range of motion of 90° without flexion contracture, Body Mass Index (BMI) < 30 kg/m^2^ [7–10]. However, these recommendations are becoming obsolete and indications are extending also to additional settings [7].

Mobile bearing (MB) and fixed bearing (FB) implants are available for PKA [8]. The polyethylene insert in FB implants is secured on the metal tibial plateau, allowing flexion, extension, and roll back [8]. On the contrary, in MB implants, the polyethylene inlay is movable on is frontal axis, allows some degree of rotation of the tibia over the femur [8]. Differences in stress distribution might lead to a difference in the outcome; however, previous evidence suggested that MB and FB promote similar outcome in PKA [8, 11]. However, to the best of our knowledge, whether MB performs better than FB implants in octogenarians has not been evaluated in a clinical setting. Therefore, a clinical trial comparing MB versus FB in PKA has been conducted. The focus of the present study was on PROMs, motion, implant positioning and implants survivorship in octogenarians. The hypothesis of the present study was that MB implants performed better than FB in PKA in octogenarians.

## Materials and methods

### Study protocol

All procedures were performed in accordance with the ethical standards of the institutional and national research committee as well as the 1964 Helsinki Declaration and its later amendments or comparable ethical standards. This study was conducted following the STROBE checklist for cohort studies [12]. Informed consent was obtained from all the participants. Appropriate ethical approval was obtained from locale ethical committee. Patient enrollment started in September 2015 and concluded in December 2019. For all the patients, the surgeries were performed by two of the authors respectively experienced in FB and MB PKA arthroplasty. Patients were not randomly allocated. The first group received FB PKA Persona Partial Knee (PPK) ® (Zimmer-Biomet, Warsaw, Indiana, USA); the second received MB PKA Oxford with Microplasty instrumentation (Zimmer-Biomet, Warsaw, Indiana, USA).

### Patient selection

The inclusion criteria were: (1) idiopathic or secondary osteoarthritis of the medial femoral compartment of the knee; (2) varus or valgus deformity < 3°; (3) knee flexion > 100°; (4) flexion contracture < 10°; (5) integrity of cruciate and collateral ligaments; (6) minimum of 24 months follow-up. The exclusion criteria were: (1) age < 80 years; (2) revision arthroplasty; (3) previous surgery of the affected knee (except meniscectomy); (3) uncontrolled systemic disease; (5) patient unable to understand the nature of the present study.

### Surgical management

Magnetic resonance imaging (MRI) was assessed pre-operatively to confirm the anatomical integrity of cruciate and collateral ligaments in all patients, and to exclude chondropathy of the lateral knee compartment. All MB-PKAs were performed with the same minimal invasive surgical approach. All the patients were placed in a supine position on a standard operating table after administering spinal anaesthesia. A tourniquet was applied to the proximal thigh on the operative side and inflated to 300 mm Hg. The operated leg was placed on thigh support with the hip flexed to about 30° degrees and the leg pendant. A medial small parapatellar incision was made; the patella was not dislocated avoiding, as much as possible, any damage to the synovial reflections of the suprapatellar pouch. The margins of the medial tibial condyle were exposed and cleared, taking care not to release too much of the soft tissue. The medial meniscus was removed. The osteophytes were removed from the tibia, femur and the intercondylar notch. The anterior cruciate ligament (ACL) status was checked. First, the tibial cut was made sagittal as near to the ACL insertion as possible. However, precautions were taken not to cut ACL fibres. The saw was parallel to the anatomical axis of the tibia and not tilted medially, laterally, anteriorly or posteriorly. The femoral cuts were then taken using the intramedullary guide and microplasty instrumentation. The flexion–extension gap balancing was measured in 20° of flexion. The extension gap is usually less than 4 mm: if the thinnest (1 mm feeler gauge) cannot be inserted, the gap is assumed to be 0 mm. Subtract the extension gap from the extension gap to calculate additional bone removal. For instance, if the extension gap measured 4 mm and the extension gap 1 mm, then the amount of bone to be milled is 3 mm. To achieve this, insert a 3 spigot and mill until the cutter will not advance further. After the final sizing, the implants were cemented and an appropriately sized mobile poly insert was used [9]. One closed suction subcutaneous drain was used and removed at first postoperative day.

All FB-PKAs were performed with the same minimal invasive surgical approach. All patients were placed in a supine position on a standard operating table after the spinal anaesthesia had been induced and the knee and the ipsilateral hip were freely mobile without the use of a tourniquet. A good exposure was obtained with skin incision starting from the medial margin of the patella to a point approximately 3 cm distal to the joint line. As reported by the surgical technique, a good ratio is usually an incision 2/3 above and 1/3 below the joint line. Further, a deep exposure was achieved with a parapatellar approach through the subcutaneous tissues to the joint capsule and the patella was laterally dislocated. A routine inspection of the patellofemoral and lateral compartment was conducted and the ACL status was checked. The medial and intercondylar osteophytes were removed, and an anterior tibial pre-cut was performed to get adequate posterior and articular view and access. In all cases, the Zimmer-Biomet PPK system (Zimmer, Warsaw, Indiana, USA) was implanted using the corresponding instrumentation, extramedullary tibial guide and femoral and tibial cutting guides. All components were cemented using Refobacin® Bone Cement *R* (Zimmer-Biomet, Warsaw, Indiana, USA). The femoral component was placed as laterally as possible. The tibial coverage was maximised without any overhang while targeting the natural tibial slope. One closed suction subcutaneous drain was used and removed at first postoperative day.

### Rehabilitation

Rehabilitation followed previous published protocols [10]. Briefly, both groups received passive mobilisation from the first postoperative day. Active progressive mobilisation of the joint and assisted walking with two crutches was started from the second postoperative day. Weight load was gradually increased during walking, and isometric muscle toning exercises were recommended until the abandonment of crutches.

### Outcomes of interest

The outcome of interest was to establish whether MB implants promote greater outcomes than FB. The range of motion (flexion and extension) was assessed using a standard logarithmic goniometer (Baseline Plastic Goniometers—Fabrication Enterprises), as reported by Hancock et al. [13], at admission (*T*_0_), at approximately 1 (*T*_1_) and 3 years (*T*_2_) follow-up. The following PROMs were administered at *T*_0_, *T*_1_, and *T*_2_: visual analogue scale (VAS) [14], Knee Society Score (KSS) [15] and Oxford Knee Score (OKS) [16]. All the assessments were conducted independently by two physicians, who were not involved in the clinical management of the patients. Data regarding the implant survivorship were also collected.

### Survivorship

Revision was defined as failure of the implant (periprosthetic joint infection [PJI], periprosthetic fracture, aseptic loosening or bearing dislocation), and survival was based on implant revision or patient death. Patient deaths were confirmed by contacting relatives. PJI was diagnosed according to the New Definition for Periprosthetic Joint Infection: From the Workgroup of the Musculoskeletal Infection Society [17]. Patients were classified as having aseptic loosening if they had symptoms including pain, instability or swelling; had radiographic evidence of loosening; and did not meet the definition for PJI [18].

### Implant positioning

Two independent observers, a trained radiologist with experience in musculoskeletal imaging and an orthopaedic surgeon, assessed implant positioning. All the measurements were performed twice with an interval of a minimum four weeks. The following parameters were measured [19]:

- *Femoral component varus/valgus* angle between the femoral component and the femoral axis in the coronal plane. Values of 7° ± 10° were considered as optimal.
- *Tibial component varus/valgus* angle between the tibial long axis and a line drawn along the tibial tray in the coronal plane. Values of 0° ± 5° were considered as optimal.
- *Anteroposterior slope* angle between a line drawn along the tibial tray and perpendicular to the tibial axis in the lateral view. Values of 7° ± 5° were considered as optimal.

All imaging were extracted from the Digital Imaging and Communications (DICOM) in Medicine data from Picture Archiving and Communications System (PACS) and inserted into OsiriX® imaging software (version 4.1.2 32-bit) [20].

### Statistical analyses

All tests were two-sided and *p*-value less than 0.05 was taken as statistically significant. Statistical analyses were conducted in *R* (version 4.1.1). An estimated sample of 48 subjects, 24 for each group, were required to compare VAS between fixed and mobile groups with a two-sided Wilcoxon–Mann–Whitney test, assuming a mean difference of 1.5, a standard deviation of 1.5 for both groups, a 5% alpha, and 90% power. Given the same parameters, this sample had also a 97% power to detect a pre-post difference using a paired Wilcoxon signed-rank test and assuming a correlation of 0.30 between measurements. Additional subjects were recruited to ensure statistical significance in case of adverse events. Summary statistics are presented as mean and standard deviation (SD) or absolute frequencies and percentages. *T*-test and odd ratio were used respectively to assess continuous or binary variables, with values of *p* < 0.05 considered statistically significant. Bonferroni adjustment was performed for multiple comparisons. Survivorship was estimated using the Kaplan Meier curve to assess differences in failure rate between fixed and mobile.

## Results

### Patient recruitment

**Fig. 1:**
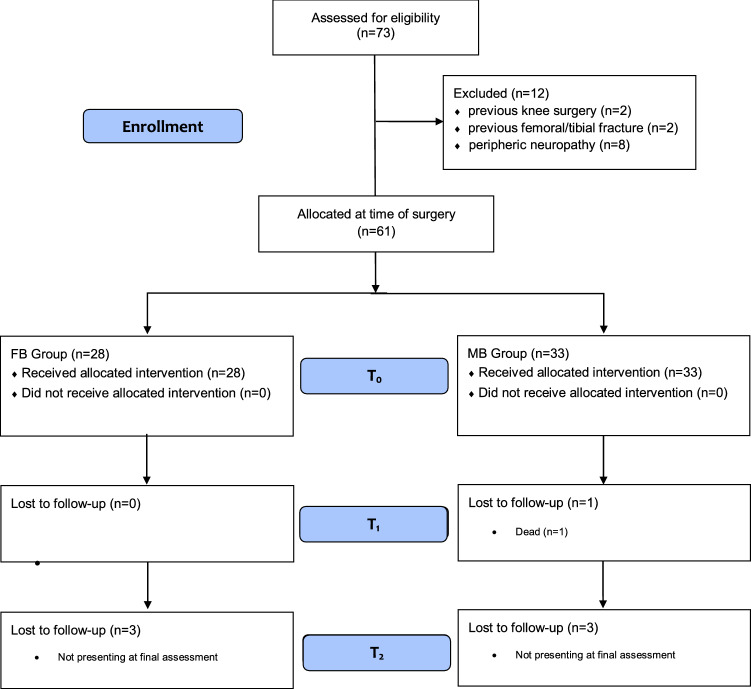
STROBE flow chart of the literature search

A total of 73 patients were initially recruited. Of them, 12 were not eligible: previous high tibial osteotomy (*N* = 2), previous femoral/tibial fracture (*N* = 2), periphery diabetic neuropathy (*N* = 8). At *T*_0_, 28 patients were included in the FB and 33 in the MB group. One patient in the MB group was lost at *T*_1_ (death). At *T*_2_, three patients were lost in each group. Finally, 25 were included in the FB group and 29 in the MB group. The STROBE diagram of the recruitment is shown in Fig. 1 [21].

### Patient demographic

Patient demographic at admission, surgical duration, and the follow-ups are reported in Table 1. Optimal comparability was observed in both groups at admission. The surgical time was shorter in the FB group (*p* < 0.001).

**Table 1:** Patient demographic

Endpoint	FB	MB	*p*-value
Mean age	82.3 ± 2.0	81.9 ± 1.0	0.5
Women*, n *(%)	23 (82%)	25 (76%)	0.8
Right side*, n *(%)	14 (50%)	14 (42%)	0.7
Surgical time	41.0 ± 6.2	48.4 ± 6.7	< 0.001*
*Pre-operatory scores*			
VAS	7.4 ± 1.2	7.4 ± 1.5	0.9
Flexion	96.6 ± 8.3	96.2 ± 8.6	0.9
Extension	3.9 ± 3.1	3.9 ± 3.3	0.9
KSS	37.3 ± 8.2	37.1 ± 9.6	0.9
Oxford Knee score	21.8 ± 3.7	21.2 ± 3.7	0.5
*Follow-up (months)*			
T_1_	12.8 ± 0.8	13.2 ± 0.8	0.09
T_2_	33.6 ± 7.4	40.8 ± 12.8	0.05

*FB* fixed bearing, *MB* mobile bearing, *VAS* visual analogue scale for pain

*Statistical significant value (*p* < 0.05)

### PROMs

No difference between FB and MB in VAS, KSS, and OKS (Table 2).

**Table 2:** Results of PROMs

Endpoint	FU	FB	MB	*p*-value
Visual Analogue Scale	*T*_0_	7.4 ± 1.2	7.4 ± 1.5	0.9
	*T*_1_	1.6 ± 1.2	1.8 ± 1.2	0.4
	*T*_2_	1.4 ± 0.9	1.5 ± 0.9	0.8
Knee Society Score	*T*_0_	37.3 ± 8.2	37.1 ± 9.6	0.9
	*T*_1_	89.6 ± 7.2	90.6 ± 5.5	0.6
	*T*_2_	90.8 ± 5.5	90.9 ± 4.9	0.9
Oxford Knee Score	*T*_0_	21.8 ± 3.7	21.2 ± 3.7	0.5
	*T*_1_	43.4 ± 2.3	43.9 ± 1.9	0.7
	*T*_2_	43.8 ± 1.7	43.8 ± 1.7	0.9

*FU* follow-up, *FB* fixed-bearing, *MB* mobile-bearing

### Range of motion

No difference was found in ROM at both follow-ups (Table 3).

**Table 3:** Results of range of motion

Endpoint	FU	FB	MB	*p*-value
Flexion	*T*_0_	96.6 ± 8.3	96.2 ± 8.6	0.9
	*T*_1_	116.4 ± 5.8	116.9 ± 4.5	0.7
	*T*_2_	117.8 ± 4.1	117.4 ± 3.9	0.7
Extension	*T*_0_	3.9 ± 3.1	3.9 ± 3.3	0.9
	*T*_1_	0.5 ± 1.6	0.6 ± 1.7	0.8
	*T*_2_	0.4 ± 1.4	0.5 ± 1.6	0.8

*FU* follow-up, *FB* fixed-bearing, *MB* mobile-bearing

### Implant positioning

No difference was found in implant positioning (Table 4).

**Table 4:** Radiographic measures at *T*_2_

Endpoint	FB	MB	*p*-value
Femoral angle	6.8 ± 4.1	6.7 ± 3.2	0.9
Tibial angle	2.6 ± 1.9	2.5 ± 2.3	0.9
Tibial slope	5.6 ± 1.7	5.7 ± 1.8	0.9

*FB* fixed-bearing, *MB* mobile-bearing

### Failure rate

**Fig. 2:**
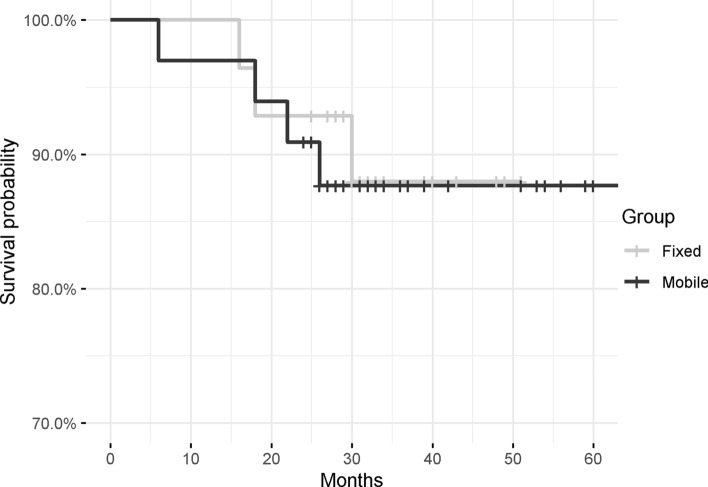
Kaplan–Meier curve

At last follow-up, FB group reported three failures caused by aseptic loosening. Four failures were observed in the MB cohort: two for bearing dislocation and two for aseptic loosening. The Kaplan–Meier Curve found no differences in implant survivorship (Fig. 2).

## Discussion

According to the main findings of the present clinical trial, MB implants performed similar to FB in PKA in octogenarians. The MB group demonstrated shorter surgical time. No difference was found in PROMs, ROM, implant positioning, and survivorship.

We were unable to identify previous clinical study which compared MB versus FB in PKA in octogenarians. Similar results were found in previous systematic reviews and meta-analyses in the younger population, which found no clinically relevant benefits of MB over the FB implants [11, 22, 23]. In a recent meta-analysis on 25 studies (4696 patients) comparing MB and FM for PKA, no difference in the outcome was found in ROM, Knee Scoring System, and its function subscale, and OKS [24]. No difference was found in the rate of revision, aseptic loosening, deep infections, fractures, and further extension of OA to the contralateral joint compartment [24]. Previous high quality clinical trials which compared MB versus FB in PKA in the younger population found similar outcomes alike. Forster et al. found no difference in function, motion and survivorship at 2 years follow-up on 30 patients [25]. Artz et al. randomly allocated 205 patents to MB versus 284 to FB [26]. At 2 years follow-up, Patient Reported Kneeling Ability was similar between the two cohorts [26]. Confalonieri et al. [27] found no difference in outcome between the two groups at 5.7 years follow-up on 40 patients. Gilmour et al. [28] randomized 58 patients to FB robotic-arm-assisted versus 54 patients who underwent conventional PKA. At 2 years follow-up, no difference was evidenced in the Knee Score Society Score and OKS [28]. Koppens et al. [29] randomly allocated 33 patients to MB Oxford PKA versus 32 patients allocated to FB Sigma PKA. At 2 years follow-up, similarity was found in OKS, RAND-36 score, and leg extension power between the two implants [29].

Osteoarthritis progression to the contralateral side, aseptic loosening, and polyethylene insert wear, are the most common reason for revision in PKA [22]. The present study found no difference between MB and FB in implant survivorship, which was consistent with previous evidence from meta-analyses [11, 22, 23]. However, three cases of aseptic loosening in the FB group and two bearing dislocations and two aseptic loosening in the MB implants were observed.

This data regarding aseptic loosening, is coherent with current literature. In fact, the primary mechanisms for UKA failure have remained consistent since early clinical reports in the 1980s. Goodfellow et al. cited aseptic loosening as the primary reason for revision (incidence 6.6%) in their series of 103 patients with an MB implant at mean follow-up of 3 years [30]. A recent systematic review found that the most common reasons for UKA failure were aseptic loosening (36%), progression of osteoarthritis (20%), unexplained pain (11%), instability (6%), infection (5%), and polyethylene wear (4%) 0.25 The majority of early failures (< 5 years) were from aseptic loosening (25%), osteoarthritis progression (20%), and bearing dislocation (17%) [31].

Insights on implants biomechanics might explain such difference in the reasons for revision. Given their more congruent bearing surfaces with a larger contact area, MB implants have been introduced to better reproduce the physiological knee kinematics [9]. Moreover, MB implants, minimizing contact stress and constraint, should reduce polyethylene wear and implant loosening, ensuring greater survivorship [9]. On the other hand, MB implants are very sensitive to soft tissue balancing, which may predispose to bearing dislocation or impingement [7]. Soft tissues undercorrection in MB might lead to higher component stress, and contribute to polyethylene dislocation; any overcorrection promotes greater contact stress in the contralateral compartment, accelerating OA progression [9]. Given its flat tibial articular surface, FB implants are easier to implant, and the risk of bearing dislocation is minimal [32]. In this respect, FB implants could offload the contralateral compartment, slowing or preventing osteoarthritis progression [32].

Regarding medial collateral ligament (MCL) tension, a computational study highlights that the implantation of an UKA to the medial side influence the distribution of load transfer in knee joint. In other words, MCL force increase keeps the knee from tilting too much into valgus during loading, thus preventing overload on the lateral side. In addition, decreased contact stress on lateral compartment in valgus model supported it. Therefore, more loads pass throughout the medial compartment even in optimum balancing condition. It may lead to clinical problems such as loosening of the tibial component or fractures of the medial tibial plateau or the underlying bone [33]

The flat tibial component of FB implants, given their fatigue and sheer stress-related mechanism, are less compliant during flexion and can lead to point loading; hence, they are more prone to inlay surface deformation and delamination [34].

The present study has several limitations. Patients were not randomly allocated. These limitations impacted negatively the quality of our conclusions, increasing the risk of selection, detection, and performance biases. However, we must point out that randomization and blinding is not easily accepted by patients and surgeons alike. The reduced sample size may jeopardize the efficacy of such study to identify seldom complication; however, given the restriction of the eligible orthopedic interventions during the Covid-19 pandemic, the patient recruitment stopped. Moreover, 26% (19 of 73) of patients were lost at follow-up. In this respect, we further remark the negative impact that the Covid-19 pandemic exert on the conduction of the present study, especially in the patient follow-up.

## Conclusion

According to the main findings of the present clinical trial, MB implants performed similar to FB in PKA in octogenarians. The FB group demonstrated shorter surgical time. No difference was found in PROMs, ROM, implant positioning, and survivorship.

## Data Availability

Raw data have been submitted as supplementary material to the Journal.
